# Impact of Dental Caries on the Quality of Life of Preschool Children and Families in Amman, Jordan

**DOI:** 10.3290/j.ohpd.a44694

**Published:** 2020-07-04

**Authors:** Lamis Darwish Rajab, Rawan Borhan Abdullah

**Affiliations:** a Professor, Department of Pediatric and Preventive Dentistry, School of Dentistry, University of Jordan. Amman, Jordan. Conceived the study and supervised the study; analysed data; wrote, read and approved the manuscript.; b Postgraduate Student, Department of Pediatric and Preventive Dentistry, School of Dentistry, The University of Jordan. Amman, Jordan. Analysed the data; read and approved the final manuscript.

**Keywords:** early childhood caries, quality of life, preschool children, Jordan

## Abstract

**Purpose:**

Early childhood caries (ECC) may have a harmful impact on quality of life (QoL) of young children and parents. No oral health-related quality of life (OHRQoL) studies had been carried out among preschool children in Jordan. The aims of the study were to assess the prevalence of ECC among preschool children and to evaluate its impact on the QoL of children and families.

**Materials and Methods:**

A cross-sectional survey was conducted among preschool children aged 4 and 5 years. A two-stage random sampling design was implemented. Parents answered the Early Childhood Oral Health Impact Scale (ECOHIS) which was used to assess OHRQoL and a questionnaire addressing sociodemographic data. Clinical examination included ECC, anterior malocclusion treats (AMT) and traumatic dental injuries (TDIs). The simultaneous influence of different independent variables including gender, AMT, TDI and socioeconomic indicators (SEI) on the overall QoL was also investigated. Analysis of variance test (ANOVA), the Fisher’s least statistically significant difference criteria of post hoc and simple logistic regression were used for statistical analysis.

**Results:**

Of the 2164 targeted preschool children, 1557 were included in the study. Prevalence of ECC was 72.5% and 77.2% among 4 and 5 year olds, respectively. Compared with caries-free children, ECC children (dmft 1–4 and dmft >4) had significantly higher mean scores of all the items of the ECOHIS (p <0.05, 0.01 and 0.001) as well as mean scores of overall ECOHIS (p <0.001). There was a significant increase in mean scores of items (p <0.05, 0.01) and overall ECOHIS mean scores (p <0.05) with increasing severity of dental caries. Only dental caries had a significant effect on ECOHIS (OR = 4, CI 3.179–5.972, p <0.001) while other confounders failed to demonstrate any impact.

**Conclusions:**

Dental caries prevalence was high and the level of severity was associated with worse OHRQoL of preschool children and families.

Preschool children can have several oral health problems, such as teething pain, eruption disturbances, early childhood caries (ECC) and dental trauma. These oral troubles can have a profound impact on the quality of children’s lives.^34^ ECC is a chronic, infectious disease affecting young children, and constitutes a serious public health problem. It is one of the most common preventable diseases and is on the rise worldwide. ECC is a multifactorial disease consequent to the interaction of cariogenic microorganisms, exposure to carbohydrates, inappropriate feeding practices, and a range of social variables.^7^ ECC is defined as the presence of one or more decayed (non-cavitated or cavitated lesions), missing (due to caries), or filled tooth surfaces in any primary tooth in a child under the age of 6 years. The definition of severe early childhood caries is any sign of smooth-surface caries in a child younger than 3 years of age, and from ages 3 through 5, one or more cavitated, missing (due to caries), or filled smooth surfaces in primary maxillary anterior teeth or a decayed, missing, or filled score of greater than or equal to four (age 3 years), greater than or equal to five (age 4 years), or greater than or equal to six (age 5 years).^13^ ECC can start early in life, progresses rapidly in high risk individuals, and often goes untreated. Its consequences can affect the immediate and long-term QoL of the children and their families and can have detrimental effects socially and financially. Children who had caries of primary dentition early in the life have a higher chance to suffer from future caries in both the primary and permanent dentitions.^11^

Traumatic injuries to the primary dentition present special problems and the management is often different as compared with the permanent dentition.^28^ Although the oral region comprises as small an area as 1% of the total body area, oral injuries account for 5% of all bodily injuries in all ages, and in preschool children the proportion is as high as 17%.^38^ Emergency situations present a challenge to clinicians worldwide. It is now recognised that child injuries are a major threat to child health and that they are a neglected public health problem.^50^

Malocclusion is a global public health problem that has an effect on how the person perceives himself and how he is perceived by the society.^47^ The level of learning ability and academic achievement of children and adolescents is highly affected by the school environment where children who are attractive are usually more socially active and friendly with others^32^ because the face is a slightly stronger indicator of overall attractiveness than the body.^33^ It was shown that having a disharmonious smile with irregular teeth and unmatched jaws exerts a huge impact on the social relationships and mental abilities of individuals which may result in reduced self- esteem and sadness due to teasing and feeling of inferiority and so affecting their quality of life (QoL).^30,46^

Recently, interest has focused on evaluating the oral health-related quality of life (OHRQoL) of children and adolescents. The concept of OHRQoL relates to the impact which oral health or disease has on the individual’s daily functioning, well-being or QoL. Oral diseases and disorders during childhood can have a negative impact on the life of preschool children and their parents.^14,34^

Due to the increase in interest in the assessment of children’s QoL worldwide, researchers have developed and tested different OHRQoL questionnaires for children aged from 6 years or older.^19-21,26^ For younger children, this category of research is limited. Evidence from the child development and psychology literature indicated that children younger than 6 years of age are unable to accurately recall every day and unique events beyond 24 h. Children begin to reason about the timing of past events with respect to the day of the week, month or season at the age of 7 or older.^44^ In addition, only at about 6 years of age do children become capable of abstract thinking, which likely underlies many perceptions of health and disease.^17^ Research that has attempted to use preschool age children as respondents in OHRQoL studies has met with limited success.^14^ The developmental characteristics of children mean that adults must report impacts of dental disease in these children.^34^ Responsibility for the health of young children is usually borne by adults. Also, adults generally make decisions about their children’s health. Therefore, assessing parents’ perceptions about how oral health problems, including symptoms, disease and its treatment influence their children’s QoL is important. The influences on caregivers also are important to measure as part of assessing young children’s OHRQoL.^34^ The Early Childhood Oral Health Impact Scale (ECOHIS) was developed and validated to assess the impact of oral health problems and related treatment experiences on the QoL of preschool age children (3–5 years old) and their families. This scale is a proxy measure that considers parents/caregivers to be fundamental in the treatment decision-making process and perceptions regarding children’s oral health conditions.^34^ Zaror et al conducted a study to obtain a systematic and standardised evaluation of the current evidence on the development process, metric properties, and administration issues of the OHRQoL instruments available for population aged 0–18 years and found that the most highly rated one was the ECOHIS in preschoolers. Among the identified questionnaires in preschool children, only the ECOHIS presented good reliability, responsiveness, and interpretability. They concluded that the evidence supports the use of the ECOHIS for preschoolers.^51^ Furthermore, the ECOHIS is the only questionnaire that has been culturally adapted to 14 languages or countries (allowing international studies) and has a section assessing the impact of oral problems on the family, making it the most complete instrument. Although the ECOHIS was originally developed to assess the impact of dental caries, it has been widely used to evaluate several oral pathologies.^1,15^

To the best of our knowledge, no studies have been conducted on the impact of ECC on the QoL of preschool children in Jordan – this study was the first. The specific aims of this study were to assess the prevalence of ECC among preschool children aged 4 and 5 years in Amman, the capital of Jordan, and to evaluate the impact of ECC and its severity on the QoL of these preschool children and their families.

## Method and Materials

### Ethical Consideration

This study received approval from the Academic Research Ethics Committee of the School of Dentistry and from the Council of the School of Postgraduate Studies at the University of Jordan. Authorisation from the Ministry of Education was also obtained for the participation of preschool children. Prior to data collection, all parents or primary caregivers who accepted their children to be recruited into the study provided a signed written informed consent form.

### Study Design and Sample Characteristics

A cross-sectional study was performed in 2015. The survey targeted preschool children of all genders aged 4 and 5 years regularly attending preschools in Amman, the capital of Jordan. A power calculation was used to determine the minimum sample size required to establish statistical significance. Using a prevalence figure of 52%,^41^ setting the confidence level at 95%, and using a margin of error of 2.5%, the minimum required sample was 1482. Preschools in Amman are either private or public. The total number of 4- and 5-year-old children who were attending preschools at the time of this survey was 11,445 and 31,578 children, respectively. Compared to public preschools, private preschools are greater in number because they were established many years earlier. At the time of the survey there were 762 private preschools with 39,718 attending children aged 4 and 5 years, and 84 public preschools with 3305 of only 5-years-old children. A two-stage random sampling design was implemented. In the first stage and to ensure representativeness, a proportional simple random sampling procedure was used to select 5% of private preschools and 10% of public preschools from different areas in Amman. Using class lists of children classified by age and gender, the second stage involved a random selection of every other class from each selected preschool. All children in the selected classrooms were included in the study. To account for contingencies such as non-response or recording error, the sample size was increased, and the total targeted sample included 2164 children. Children whose parents did not sign the consent form or were absent the day of clinical examination, uncooperative and highly anxious, and those with a medical condition, on regular medications or with special health needs were excluded from the study.

### The Questionnaires

Prior to data collection, all parents or primary caregivers allowed their children to be recruited into the study filled in a questionnaire which included questions about sociodemographic data, as well as the ECOHIS.

Sociodemographic information included data on the child’s age, gender, socioeconomic indicators (SEI), medical status of the child and parent opinion about their child’s general and dental health (good, poor).

To measure the OHRQoL, the study used the ECOHIS. It was developed by Pahel et al to measure the OHRQoL for children younger than 6 years.^34^ It was translated into Arabic and its validity and reliability were tested by Pani et al.^35^ It contains 13 questions corresponding to four descriptive domains for items included in the child impact section: symptoms, function, psychological and self-image and social interaction. The family impact section has two domains: parental distress and family function.^34^ Response categories for the ECOHIS are coded: 0 = never; 1= hardly ever; 2 = occasionally; 3 = often; 4 = very often and 5 = don’t know. ECOHIS scores are obtained by simple summation of the response codes. The total score ranges between 0 and 52, with a higher ECOHIS meaning a poorer OHRQoL.^34^

### Calibration Exercise

The clinical oral examination of the children was performed by a single dentist (RA) who underwent a calibration exercise prior to the study. The results of the examinations were compared with the judgment of an experienced paediatric dentist (LDR) in the diagnosis of dental caries and traumatic dental injuries (TDIs). There was high interexaminer agreement for dental caries and TDI. The Kappa score was 0.97 for dental caries, and 0.95 for TDI. During data collection, a group of 50 children were re-examined 2 weeks after the initial examination under the same conditions and a high intraexaminer Kappa value of 0.94 for dental caries and 0.96 for TDI were obtained indicating excellent agreement.

### Pilot Study

A pilot study was conducted prior to the main survey on a sample of 25 preschoolers (4 and 5 years of age) and their parents/caregivers – who were not included in the main study – to test the design, the clinical examination and administration of the questionnaires. The results showed that the readability, parents’ ease of interpreting the questionnaires, and the self-administered format of the questionnaires were satisfactory. The results of the pilot study indicated there was no need to change the proposed methods.

### Clinical Oral Examination

Examination was carried out in the nursery/kindergarten medical room, if one was available, or in the classroom. The dental examination was carried out using disposable gloves, disposable examination set for each participant (mirror, World Health Organization (WHO) mouth probe and tweezer), ruler, and torchlight to aid illumination. The child sat on a static chair situated in the room where the light would aid illumination supplemented with the torchlight. Universal infection control precautions were followed during the examination.

### Dental Caries

ECC was diagnosed based on the standardised criteria of the WHO,^49^ which has been mainly made visually. No radiographs were used. ECC was calculated in terms of decayed, missing due to caries and filled primary teeth (dmft). Then dmft was categorised according to the severity of ECC based on a previously proposed scores^9^: dmft 0 = caries free, dmft 1–4, and dmft >4.

### Anterior Malocclusion Traits

The anterior malocclusion traits (AMT) used by Abanto et al was adopted.^1^ It is the most commonly malocclusion classification used in the preschool age in which the three most common AMT found at preschool children are assessed: anterior open bite, overjet ≥ 4 mm and anterior cross bite. If at least one of these AMT is present, the child is categorised as having malocclusion.^1^

### Traumatic Dental Injuries (TDIs)

The epidemiological classification adopted by the WHO and modified by Andreasen et al was used to record TDI.^6^ The child was considered having TDI when at least one kind of trauma was present regardless of its type or if there is tooth discoloration.

### Socioeconomic Indicators

Finding an accurate definition of socioeconomic class is still a problem and class structure in Jordan is exceedingly difficult to assess. However, based on a previously conducted study in Jordan^45^ and on others^1,3,23,40,48^ to allow international comparison, the socioeconomic status of the parents was assessed using three variables: mother’s education (secondary education, college diploma, and university degree or higher), mother’s employment (employed, unemployed), and monthly tuition fees paid for preschool by Jordanian Dinar (JD) (free of charge, 50 to <100 JD, 100–200 JD).

### Data Analysis

Analysis was performed using the Statistical Package for the Social Sciences (IBM-SPSS for Windows, version 24.0, SPSS, and Chicago, IL, USA). Each group of children with caries (dmft 1–4) and (dmft >4) was compared to children who were caries free. Gender, AMT, TDI, and SEI were used as independent variables. TDI was dichotomised into absent or present. Each trait of the AMT was dichotomised into absent or present. Kappa test was used to test inter and intraexaminer reliability. Data analysis involved descriptive statistics for demographic characteristics (frequency distribution and cross tabulation). Analysis of variance test (ANOVA) was used to calculate means and to determine the statistically significant impact of absence of caries, dmft 1–4 and dmft >4 on each item of ECOHIS, on ECOHIS domains and on overall ECOHIS. Statistically significant differences were compared across the groups using the Fisher’s least statistically significant difference criteria of post hoc analysis.

Logistic regression analysis with a stepwise selection procedure was used to investigate the simultaneous influence of different independent variables (gender, AMT, TDI, and SEI) on the overall QoL. All variables were included at the start and those failing to show a statistically significant relationship in the univariate analysis were not considered for the multivariate analysis. For the purpose of using binary logistic regression, a new dependent variable was created: impact on overall ECOHIS. This dependent variable has been dichotomised into absent and present, assuming the distinctive differences related to existed and none existed trait. Dental caries was also dichotomised and served as well as the confounders (gender, AMT, TDI, and SEI) as independent variables. The level of statistical significance for all tests was set at 5%.

## Results

Of the 2164 targeted preschool children, 1929 returned positive consent with a response rate of 89.1% (89.4% for private and 88% for public preschools). Parents who refused their children’s participation in the study justified that they were afraid about their young children experiencing a psychological trauma from being examined without their presence. At the day of examination, 272 preschoolers were absent (174 from private and 89 from public preschools) and thus only 76.6% (1657) were examined. However, 87 children were excluded from the study (34 from private and 53 from public preschools) as their parents answered ‘don’t know’ to one or more items of the ECOHIS and 13 children (10 from private and 3 from public preschools) because they had systemic diseases. The final number of the included children in the study was 1557 (1358 from private and 199 from public preschools) which represented 72% of the targeted sample (77% for private and 49.6% for public preschools). The demographic characteristics of the study sample and information about child’s dental health are presented in Table 1.

**Table 1:** Parent and child demographic characteristics in the study sample (n = 1557)

Variable			N	%
Mother	Mother’s educational level	Secondary education	467	30.0
		College diploma	306	19.6
		University degree or higher	784	50.4
	Mother’s employment status	Employed	1009	64.8
		Unemployed	548	35.2
Child	Age	4 years	404	25.9
		5 years	1153	74.1
	Gender	Male	775	49.8
		Female	782	50.2
	Kindergarten type	Public	199	12.8
		Private	1358	87.2
	Monthly tuition fees	Free of charge	199	12.8
		50 to <100 JD	600	38.5
		100–200 JD	758	48.7
Child’s general and dental health status	General health rating	Good	1555	99.9
		Poor	2	0.1
	Dental health rating	Good	1334	85.7
		Poor	223	14.3

Table 2 shows the prevalence rate and caries experience of preschool children by age and gender. In both ages, males had significantly higher caries experience than females (p = 0.000). The prevalence of dental caries among 5-year-olds was significantly higher than that found among 4-year-olds (p <0.039). At the age of 4, 41% (165) had dmft = 1–4 and 32% (128) had dmft >4. For 5-year-old, 48% (553) had dmft = 1–4 and 29% (128) had dmft >4. Of the examined children, 34.5% (537) had AMT. The majority of preschool children had not experienced TDI with a prevalence of only 3.2% (50).

**Table 2:** Caries prevalence rate (%) and caries experience of preschool children (dmf-t) according to age and gender

Indicator	Males	Females	Total
4 years	(n = 209)	(n = 195)	(n = 404)
Prevalence	75.1%	70%	72.5%
dmf-t	3.9***	3.2	3.6
			
5 years	(n = 566)	(n = 587 )	(n = 1153)
Prevalence	77.0%	77.3%	77.2%
dmf-t	4.1***	3.6	3.9

***p <0.001.

The responses to the ECOHIS items are presented in Table 3. In the child impact section, ‘pain’ was the most frequently reported item (45.4%), followed by ‘eating’ (28.3%), and ‘irritable or frustrated’ (27.2%). Items related to ‘feeling guilty’ (23.9%), or ‘feeling upset’ (19.0%) and ‘financial impact to the family’ (19.0%) were the most reported in the family impact section of the ECOHIS.

**Table 3:** Distributions of the ECOHIS responses (n = 1557)

	Oral health-related quality of life item (ECOHIS)	Never or hardly ever	Occasionally, often, or very often
		N (%)	N (%)
1	Oral/dental pain	850 (54.6)	707 (45.4)
2	Difficulty drinking	1212 (77.8)	345 (22.2%)
3	Difficulty eating	1117 (71.7)	440 (28.3)
4	Difficulty pronouncing words	1302 (83.6)	255 (16.4)
5	Missed preschool	1374 (88.2)	183 (11.8)
6	Trouble sleeping	1300 (83.5)	257 (16.5)
7	Irritable or frustrated	1133 (72.8)	424 (27.2)
8	Avoided smiling or laughing	1404 (90.2)	153 (9.8)
9	Avoided talking	1432 (92.0)	125 (8.0)
10	Been upset	1262 (81.0)	295 (19.0)
11	Felt guilty	1185 (76.1)	372 (23.9)
12	Time off from work	1369 (88.0)	188 (12.0)
13	Financial impact	1259 (81.0)	298 (19.0)

Table 4 provides the descriptive statistics of the ECOHIS responses: ranges; floor effect (proportion with score of 0); mean and standard deviation values. No impacts (floor effects, ie, the lowest possible score of 0) were reported by 9% of parents on the child impact section (91% reported that their child experienced at least one oral health impact) and 47.5% on family impact section (52.5% reported that the family was affected as a result of their child’s oral health). Floor effects were immense on the ‘self-image and social interaction’ (76.9%), and ‘child psychology’ (49.4%) in the child impact section, and with respect to parental distress (58.7%) and family function (63.0%) in the family impact section. For overall ECOHIS, 8.2% had reported no impact. No maximum effects were observed for either of the two sections (ie, scores of 36 and 16 on the child and family impact sections, respectively). The maximum number of impacts reported was 12 on the child impact section and 8 on the family impact section. Table 4 also indicates that parents reported more child impacts than family impacts. The mean scores of overall ECOHIS in the child impact section was higher (5.6) than that in the family impact section (2.1). In the child impact section, the child function domain had the highest mean score (2.4) followed by child symptoms (1.3). The least mean score (0.6) was of child self-image and social interaction domain. In the family impact section, the parental distress domain had a slightly higher mean (1.2) than family function domain (0.9); the QoL in the family section was mostly equally affected by these domains.

**Table 4:** Descriptive distributions of the ECOHIS responses for different domains and overall (n = 1557)

Variables	N of items	Possible range	Range	Floor effect N (% score 0)	Mean (SD)
**Overall child impact section**	9	0–36	0.0–27.0	140 (9.0)	5.6 (4.9)
Child symptoms domain	1	0–4	0.0–4.0	448 (28.8)	1.3 (1.1)
Child function domain	4	0–16	0.0–12.0	493 (31.7)	2.4 (2.5)
Child psychology domain	2	0–8	0.0–8.0	767 (49.3)	1.3 (1.6)
Child self-image and social interaction domain	2	0–8	0.0–7.0	1197 (76.9)	0.6 (1.2)
					
**Overall family impact section**	4	0–16	0.0–16.0	739 (47.5)	2.1 (2.8)
Parental distress domain	2	0–8	0.0–8.0	914 (58.7)	1.2 (1.7)
Family function domain	2	0–8	0.0–8.0	981 (63.0)	0.9 (1.5)
					
**Overall ECOHIS items**	13	0–52	0.0–39.0	128 (8.2)	7.7 (7.0)

For each of the 13 items of the ECOHIS, mean, standard deviation, statistical significance in relation to dental caries were calculated and presented in Table 5. The dental caries groups (dmft 1–4 and dmft >4) had higher means of all the items of ECOHIS than those of the caries-free group. In both of the caries groups (dmft 1–4 and dmft >4) ‘oral/dental pain’ had the highest mean score followed by ‘irritable or frustrated’ and ‘difficulty eating’. ANOVA showed a statistically significant difference in the mean of each item between groups (p <0.05) except # 3 ‘difficulty eating’, and # 4 ‘difficulty pronouncing words’ (p >0.05). The post hoc analysis showed a statistically significant difference (p <0.05) between caries free and dmft >4 in all items. Table 5 also shows a statistically significant difference (p <0.05) in the mean scores of all items between caries-free and dmft 1–4 groups in the child impact section except #6 ‘trouble sleeping’, #7 ‘felt irritable or frustrated’, #8 ‘avoided smiling or laughing’, and #9 ‘avoided talking’, and in the family impact section #10 ‘been upset’, #12 ‘time off from work’, and #13 ‘financial impact’. Table 5 demonstrates that the child’s OHRQoL was affected by the severity of dental caries. A statistically significant difference (p <0.05) was found in mean scores between dmft 1–4 and dmft >4 groups of item #3 ‘pain’, #6 ‘trouble sleeping’, #7 ‘irritable or frustrated’, and #8 ‘avoided smiling or laughing’ in the child impact section, while in the family impact section no statistically significant difference was found between mean scores of any item (p >0.05).

**Table 5:** ECOHIS items, mean score, standard deviation, and differences between groups according to dental caries

	Oral health-related quality of life item (ECOHIS)	dmft = 0	dmft >0		ANOVA	Post hoc statistically significant difference between groups
			dmft 1 – 4	dmft >4		
		(1) n = 374	(2) n = 639	(3) n = 544		
		Mean (SD)			P	P
1	Oral/dental pain	0.98 (0.85)	1.33 (1.02)	1.49 (1.19)	0.000	2>1 (0.000) 3>1 (0.000) 3>2 (0.007)
2	Difficulty drinking	0.57 (0.8)	0.7 (0.93)	0.78 (0.96)	0.003	2>1 (0.030) 3>1 (0.001)
3	Difficulty eating	0.71 (0.94)	0.81 (1.03)	0.87 (1.07)	0.053	Not statistically significant
4	Difficulty pronouncing words	0.48 (0.83)	0.52 (0.9)	0.49 (0.86)	0.744	Not statistically significant
5	Missed preschool	0.33 (0.65)	0.43 (0.74)	0.44 (0.76)	0.045	2>1 (0.033) 3>1 (0.020)
6	Trouble sleeping	0.42 (0.74)	0.5 (0.85)	0.61 (0.95)	0.004	3>1 (0.001) 3>2 (0.033)
7	Irritable or frustrated	0.69 (0.91)	0.72 (1)	0.89 (1.04)	0.003	3>1 (0.003) 3>2 (0.004)
8	Avoided smiling or laughing	0.25 (0.6)	0.28 (0.67)	0.39 (0.76)	0.004	3>1 (0.003) 3>2 (0.008)
9	Avoided talking	0.19 (0.53)	0.26 (0.67)	0.3 (0.7)	0.039	3>1 (0.011)
10	Been upset	0.44 (0.83)	0.56 (1.01)	0.63 (0.98)	0.015	3>1 (0.004)
11	Felt guilty	0.52 (0.89)	0.66 (1)	0.71 (1.05)	0.012	2>1 (0.031) 3>1 (0.003)
12	Time off from work	0.26 (0.64)	0.34 (0.77)	0.42 (0.81)	0.006	3>1 (0.001)
13	Financial impact	0.45 (0.86)	0.56 (0.99)	0.64 (1)	0.017	3>1 (0.004)
	Overall ECOHIS	6.28 (6.36)	7.65 (7.09)	8.64 (7.16)	0.000	2>1 (0.002) 3>1 (0.000) 3>2 (0.015)

The mean scores of overall ECOHIS was significantly higher in children with caries (dmft 1–4 and dmft >4) than those in caries-free children (p <0.05 for all items). The post hoc analysis demonstrated that the more the severity of dental caries the more the negative effect on overall ECOHIS. A statistically significant difference between means of caries-free group and both dmft 1–4 and dmft >4, as well as between means of dmft 1–4 and dmft >4 groups was found (p <0.05) (Table 5).

The simple logistic regression analysis of the confounding variables (gender, AMT, TDI and SEI) and the independent variable (dental caries) showed that only dental caries had a statistically significant impact on overall ECOHIS (p = 0.000) while the other confounders failed to demonstrate a statistically significant effect (Table 6). There was no need to run the multivariate adjusted logistic regression test.

**Table 6:** Logistic regression analysis of the confounding variables (gender, AMT, TDI, SEI) and the independent variable (dental caries) according to impact on overall ECOHIS (dependent variables)

Independent variables		Impact on overall ECOHIS QoL		Odds ratio (95% CI)	P
		No impact	Has impact		
Gender	Male	56	719	0.809 (0.552–1.185)	0.275
	Female	72	710		
AMT	Present	31	506	0.679 (0.439–1.051)	0.082
	Absent	97	923		
TDI	Present	5	45	1.219 (0.452–3.284)	0.696
	Absent	123	1384		
Dental caries	dmft = 0	85	289	4.357 (3.179–5.972)	0.000
	dmft 1–4	31	608		
	dmft >4	12	532		
Mother’s education	Secondary education	25	442	0.913 (0.712–1.170)	0.470
	College diploma	31	275		
	University degree or higher	72	712		
Mother’s employment	Employed	85	924	1.181 (0.766–1.822)	0.451
	Unemployed	43	505		
Monthly tuition fees	Free of charge	14	185	1.184 (0.878–1.595)	0.268
	50 to <100 JD	42	558		
	100–200 JD	72	686		

## Discussion

This study evaluated the impact of ECC on OHRQoL of 4–5-year-old Jordanian preschool children and their families in Amman. To the best of our knowledge, this study was the first to assess the impact of dental caries on preschool children using the ECOHIS in a Jordanian population-based sample. The design of the study was cross-sectional. The sample was selected randomly to be representative of the 4–5 years population and the two types of preschools (private and public) in Amman. The design of the study provided a valid estimation of the prevalence of dental caries and its effect on OHRQoL, therefore, generalisation of the results could be done easily to the population under investigation. The response rate based on the final number of preschoolers included in the study was 72%, which is considered good.

The present study demonstrated that ECC was a significant health problem among preschool children in Amman. The majority of the 4–5-year-old preschool children were affected by dental caries, with about one-third of both ages having dmft >4. This emphasises the importance of education and promotion of oral health programmes for parents, preventive programmes for preschool children, and improvement of access to dental care to enable preventive strategies to be implemented.

The majority of parents reported that their child experienced at least one oral health impact, mostly pain and functional impairments. An effect on the family as a result of the child’s oral health was reported by about one half of parents. A small percentage of the parents reported no impact of oral health problems on the child leading to insubstantial floor effects. This finding is in line with the finding of Peker et al^36^ but lower than that reported by Pahel et al.^34^ The low floor effect found in the child section in the current study probably indicated that children genuinely had high levels of problems. The floor effect for ECOHIS appears to be in accordance with the disease characteristics of the study sample, wherein a high percentage of the sample had dental disease. Also, participants in our study were selected from a community-based sample for a cross-sectional survey conducted at preschools and were not seeking dental treatment; whereas children who were part of the Pahel et al^34^ study were from clinically based convenience samples and therefore already exhibited some type of oral health problem. No ceiling effect was noticed and this was consistent with the results of previous studies.^34,36^

The most frequently described items in the two sections in the ECOHIS of the scale were almost the same as those reported in previous studies.^24,34,36^ In our study, in the child impact section, the most prevalent items were related to ‘pain’, ‘eating’, and ‘irritable or frustrated’. ‘Feeling guilty’, or ‘feeling upset’ and ‘financial impact to the family’ were those most reported in the family impact section.

Preschool children of this study suffered more impacts than their families; this was shown by the higher mean scores of overall ECOHIS in the child impact section than that in the family impact section. Barbosa et al suggested that as oral diseases and disorders are likely the most severe and have required clinical care since birth, it could be that the parent–child relationship is to some extent closer when children have these conditions, so that parents are more accustomed with their activities and feelings.^8^

In the child impact section, the child function domain was the most affected followed by child symptoms and child psychology. The social interaction domain was the least affected and this was expected because according to the psychological development of children, those aged less than 6 years usually lack abstract thinking and self-image concept. This may explain the low mean scores of responses in the child self- image/social interaction domain.^17^ The present study demonstrated that the oral health of young children mostly affected their daily activities such as eating, drinking and speech, while it has almost no impact on their socialisation represented by smiling or talking. These results are in line with those of previous studies.^22,24,31,36,40,43^ In the family section, impact was a slightly higher on the parental distress domain than on the family function domain. This result was confirmed in other previous studies conducted among preschool children which showed that the parental distress domain was the mostly affected.^1,23,34,41^ Several studies also showed an impact on the family function domain and this impact was explained by the absence from work for caregivers who had to stay home to take care of their child or spend money in accessing dental care.^1,2^ Moreover, there was strong evidence that parents or caregivers of young children experienced significant QoL issues because of their children’s health problems and treatment experiences.^26^

The results of the presents study are alarming as they showed that ECC had high negative impact on the QoL of preschool children. ‘Oral/dental pain’, ‘irritable or frustrated’, and ‘difficulty eating’ had the highest impacts in the child section. These are symptoms frequently related to ECC. In comparison with caries-free children, all items of the ECOHIS in caries groups were significantly affected except ‘difficulty eating’ and ‘difficulty pronouncing words’. Naidu et al found that the odds for children who had difficulty eating were greater for those with dental caries than the odds ratios for children who had no difficulty eating.^31^ This difference in results might be related to type of cut-off point used in the statistical analysis; in our study we used dmft = 0, dmft = 1–4, and dmft >4 whereas Naidu et al used dmft = 0 and dmft >0.

When caries-free and dmft 1–4 groups were compared in the family impact section, ‘felt guilty’ was the only item that showed a statistically significant difference. Abanto et al reported that parents of preschool children related to them feel guilty more frequently because of their children’s dental caries.^1,2^ As the principal causes of dental caries are readily explained by oral health experts, parents know that dental caries is frequently related to sweetened food and poor oral hygiene. Amin et al suggested that parents felt guilty as they fear being blamed for the problem.^5^ Rajab et al reported that although the level of dental knowledge of Jordanian parents was high,^42^ a discrepancy between knowledge and practices in dental care was documented. For example, dental visits by children were mostly prompted by symptoms or problems with teeth. This may also explain why the other items in the family section did not show statistical significance, since few carious lesions may not be accompanied by continuous symptoms to the extent of disturbing the comforting routine of parents’ life.

This study demonstrated that the increase in the severity of ECC was associated with lower QoL. A significant poorer QoL related to all the items in both child and family impact sections was found in dmft >4 group compared to the caries-free group. The same result was found by Abanto et al and this was expected as increasing severity of dental caries means increasing symptoms which in turn will affect QoL of children and families in all life aspects negatively.^1^ Feelings of depression and disappointment are usually the dominant emotions when parents see their children suffering from painful dental caries. Moreover, in the family impact section, similarly as reported by Abanto et al, the items ‘time off from work’ and ‘financial impact’ did not have high percentages as frequent responses; however, they displayed a negative impact on OHRQoL. This may occur because most of the responses were focused in the ‘often’ and ‘very often’ options, thus decreasing the ECOHIS score in the items of family function domain.^1^

Children with dmft >4 had a significantly higher impacts related to the items ‘pain’, ‘trouble sleeping’, ‘irritable or frustrated’, and ‘avoided smiling or laughing’ when compared with children with dmft 1–4 which implies that the child’s QoL is affected by the severity of dental caries. In the family impact section, no significant difference was observed between the two caries groups. This does not mean that parents are not concerned by the oral health of their children, because when we think about dental caries, it is expected that parents had been upset and felt guilty for their child’s dental health. Our finding might lead one to think that the degree to which the parents in this study population were affected is the same whether the child had 1–4 carious teeth or more. Certainly, caries may be detected in the early stages in which only preventive measures are necessary, and the number of carious teeth does not affect the parents’ QoL unless associated with symptoms. Although some authors reported that the perception of parents/caregivers regarding their children’s oral health is influenced only by the occurrence of symptoms, such as toothache,^10,15^ Abanto et al found a significant association between parents that had been upset and felt guilty and the severity of dental caries in preschool children.^1,2^ They clarified their finding by the concept of having good oral health believed generally by the parents is when the child does not feel pain or discomfort. This explains the association of upset and guilt with dental caries, where greater severity causes more pain felt by the child, which in turn causes increased feelings of upset and guilt by the parents.^1,2^

The results of the present study showed that dental caries adversely affected the overall QoL of children and their families; impact on the overall ECOHIS of children with ECC was significantly higher than that of children who were caries free. This is likely because dental caries, which may cause pain and discomfort to the child, therefore affects family activities and emotions. This result corroborates with results of previous studies, which found that children with caries had higher mean ECOHIS scores than the caries-free children and that caries group had more impact on the overall ECOHIS.^12,22,24-25,29^ A systematic review carried out to verify whether dental caries is negatively associated with OHRQoL pointed to that all studies that assessed dental caries reported a negative association between dental caries and OHRQoL.^16^ Moreover, it had been shown that children suffering from dental caries had low QoL and their overall OHRQoL score improved after treatment under general anesthesia.^18^ Also, the present study demonstrated that the more the severity of caries the more the negative effect on overall ECOHIS. Our result was in agreement with results of previous studies which reported that there was clear and significant increase in the overall ECOHIS score with increasing severity of dental caries indicating poorer QoL of preschoolers and their parents.^1,43^ This observation could be explained by the apparent increase in symptoms due to the carious lesions interfering with daily activities of both children and their parents.

In the present study, only dental caries had a significant effect on ECOHIS while gender, AMT, TDI, and SEI failed to demonstrate a statistically significant effect on overall ECOHIS. The results of the present study were in line with results of several studies which showed that neither malocclusion nor TDI had an effect on QoL of preschool children.^1-3^ A potential explanation is partly due to the fact that TDI was recorded as present or absent, so it was dichotomised.^1^ Also it may be attributed to the low prevalence of TDI found in this study and to that, most TDI found in children were not severe injuries. Moreover and regarding malocclusion, ECOHIS is more suitable to measure the impact of dental caries and TDI on QoL rather than malocclusion.^1^ However, Perazzo et al found that having experienced a traumatic dental injury and having a malocclusion were associated with a poorer OHRQoL of preschool children.^37^ Also, Abanto et al found that increased overjet was associated with worse OHRQoL in 5-year-old children.^4^ To explain the difference between results of studies, Locker proposed that the association between oral disease and health-related QoL outcomes is mediated by personal and environmental variables.^27^

The association between sociodemographic characteristics and OHRQoL is not clear-cut.^48^ Our results had not demonstrated a significant impact of the SEI on the OHRQoL and this was in line with the results observed by other authors.^23,40^ However, these results were against those reported in other studies where a negative impact of the SEI on OHRQoL was demonstrated. Parents’ who have a lower socioeconomic status were more likely to rate their child‘s oral health ‘worse than other children.^3,39,43^

The present study has limitations inherent to the cross-sectional design and the use of questionnaires that will possibly have been subject to information bias. Using validated questionnaire and a representative sample may lessen the effects of these limitations. A large preschool population-based, epidemiological sample representative of the city of Amman (the capital of Jordan) was obtained, which permits extrapolating the findings to the general population. Another limitation is that ECOHIS is a proxy measure and caution must be exercised when interpreting data obtained from a proxy. Proxy reports on children’s oral health may underestimate the severity of oral health impacts, particularly in relation to social and emotional impacts.^8^

## Conclusions

The prevalence of ECC was high among preschool children in Amman. Children with ECC suffered a significant negative impact on all the items and overall ECOHIS. The severity of ECC significantly worsens the QoL. Only dental caries had a significant effect on ECOHIS; neither gender, AMT, TDI nor SEI had effect on QoL of preschool children.
